# A_*2*_ Adenosine Receptors Mediate Whole-Body Insulin Sensitivity in a Prediabetes Animal Model: Primary Effects on Skeletal Muscle

**DOI:** 10.3389/fendo.2020.00262

**Published:** 2020-04-28

**Authors:** Joana F. Sacramento, Fátima O. Martins, Tiago Rodrigues, Paulo Matafome, Maria J. Ribeiro, Elena Olea, Silvia V. Conde

**Affiliations:** ^1^CEDOC, NOVA Medical School, Faculdade de Ciências Médicas, Universidade Nova de Lisboa, Lisbon, Portugal; ^2^Faculty of Medicine, Institute of Physiology and Institute of Clinical and Biomedical Investigation of Coimbra (iCBR), University of Coimbra, Coimbra, Portugal; ^3^Escola Superior de Tecnologia da Saúde, Departmento de Ciências Complementares, Instituto Politécnico de Coimbra, Coimbra, Portugal; ^4^Departamento de Bioquímica y Biología Molecular y Fisiología, Facultad de Medicina, CSIC, Ciber de Enfermedades Respiratorias, CIBERES, Instituto de Biología y Genética Molecular, Instituto de Salud Carlos III, Universidad de Valladolid, Valladolid, Spain

**Keywords:** adenosine, adenosine receptors, insulin resistance, insulin signaling, adipose tissue, skeletal muscle, gender differences

## Abstract

Epidemiological studies showed that chronic caffeine intake decreased the risk of type 2 diabetes. Previously, we described that chronic caffeine intake prevents and reverses insulin resistance induced by hypercaloric diets and aging, in rats. Caffeine has several cellular mechanisms of action, being the antagonism of adenosine receptors the only attained with human coffee consumption. Here, we investigated the subtypes of adenosine receptors involved on the effects of chronic caffeine intake on insulin sensitivity and the mechanisms and sex differences behind this effect. Experiments were performed in male and female Wistar rats fed either a chow or high-sucrose (HSu) diet (35% of sucrose in drinking water) during 28 days, to induce insulin resistance. In the last 15 days of diet the animals were submitted to DPCPX (A_1_ antagonist, 0.4 mg/kg), SCH58261 (A_2A_ antagonist, 0.5 mg/kg), or MRS1754 (A_2B_ antagonist, 9.5 μg/kg) administration. Insulin sensitivity, fasting glycaemia, blood pressure, catecholamines, and fat depots were assessed. Expression of A_1_, A_2A_, A_2B_ adenosine receptors and protein involved in insulin signaling pathways were evaluated in the liver, skeletal muscle, and visceral adipose tissue. UCP1 expression was measured in adipose tissue. Paradoxically, SCH58261 and MRS1754 decreased insulin sensitivity in control animals, whereas they both improved insulin response in HSu diet animals. DPCPX did not alter significantly insulin sensitivity in control or HSu animals, but reversed the increase in total and visceral fat induced by the HSu diet. In skeletal muscle, A_1_, A_2A_, and A_2B_ adenosine receptor expression were increased in HSu group, an effect that was restored by SCH58261 and MRS1754. In the liver, A_1_, A_2A_ expression was increased in HSu group, while A_2B_ expression was decreased, being this last effect reversed by administration of MRS1754. In adipose tissue, A_1_ and A_2A_ block upregulated the expression of these receptors. A_2_ adenosine antagonists restored impaired insulin signaling in the skeletal muscle of HSu rats, but did not affect liver or adipose insulin signaling. Our results show that adenosine receptors exert opposite effects on insulin sensitivity, in control and insulin resistant states and strongly suggest that A_2_ adenosine receptors in the skeletal muscle are the majors responsible for whole-body insulin sensitivity.

## Introduction

In the past decades the prevalence of lifestyle diseases associated with metabolic disturbances like insulin resistance and obesity, core features in type 2 diabetes, has increased. Prediabetes and type 2 diabetes are characterized by defects in insulin secretion and insulin resistance, which leads to a decrease in whole-body glucose disposal (1). These diseases, seen as men's illness for decades, presently are more common in women than in men, and obesity has a higher association to insulin-resistance related risk factors in women than in men. Females tend to be more obese than men (2) with more women being overweight or obese after the age of 45 year, being this correlated with the higher incidence of insulin resistance and type 2 diabetes in females (2). Genetic effects and epigenetic mechanisms, nutritional factors, and sedentary lifestyle affect risk and complications differentially affect males and females and may be in the origin of sexes differences regarding metabolic diseases (3). Therefore, the study of the differential mechanisms on overall insulin sensitivity and metabolism between sexes may contribute to fill the gap in the current knowledge on sex-driven mechanisms, with a major impact for personalized medicine and societal strategies.

Caffeine is the most widely behaviorally active substance consumed in the world and when consumed chronically appears to have minor negative consequences on human health (4). Several epidemiological studies showed that chronic caffeine intake decreases the risk of type 2 diabetes in men and women (5–7). Additionally, our group described that chronic caffeine intake prevents the development of insulin resistance in female and male rats with prediabetes induced by the hypercaloric diets (8) and reversed insulin resistance in aged rats (9).

Caffeine has several mechanisms of action at a cellular level, however the only mechanism achieved with regular human caffeine consumption is the antagonism of adenosine receptors (4). Adenosine is a product of ATP catabolism, which can be recycled to re-synthesize ATP itself and exerts its action through four different G-protein coupled receptors, A_1_, A_2A_, A_2B_, and A_3_ (10). This mediator is involved in key pathways that regulate glucose homeostasis and insulin sensitivity, however its role remains controversial. Adenosine has been described to be associated with insulin sensitivity and glucose tolerance via action on adenosine A_1_ receptors (11). In contrast, Figler et al. (12) showed that adenosine through A_2B_ adenosine receptors was involved in insulin resistance and inhibited whole body glucose disposal. In skeletal muscle, *in vitro* studies described an inhibitory effect of adenosine on glucose utilization and glucose transport induced by insulin (13–15), an effect that was shown to be mediated by A_1_ adenosine receptors (16). In contrast, other *in vitro* studies in skeletal muscle showed that adenosine has a stimulatory effect of insulin-induced glucose transport via A_1_ adenosine receptors (17–19).

In isolated rat hepatocytes, activation of A_1_ adenosine receptors triggers glycogenolysis, whereas the activation of adenosine A_2A_ receptors increased gluconeogenesis (20). In contrast, other studies showed that the stimulation of adenosine A_2B_ receptors augments glycogenolysis and gluconeogenesis (21, 22). In adipose tissue, it is consensual that adenosine inhibits lipolysis and stimulates lipogenesis through A_1_ adenosine receptors (23–27). This is in agreement with the increase in lipolysis, fat oxidation, and thermogenesis observed with caffeine intake and which contribute to its protective role in type 2 diabetes (28–30).

There is accumulating evidences from animal and human studies showing that central sympathetic overactivity plays a pivotal role in the etiology and complications of insulin resistance (31, 32). Activation of sympathetic nerves innervating the liver produce a rapid and marked production of glucose following a meal but promotes gluconeogenesis when fasted; and adrenal medulla activation can also stimulate the release of catecholamines to promote hepatic glucose production [for a review see Conde et al. (33)]. Sympathetic nerves innervating the skeletal muscle can promote glucose uptake independently of insulin through activation of β-adrenergic receptors, an effect counteracted by the neuronal stimulation of α-adrenergic receptors in arterioles, which elicits vasoconstriction (33). Acute caffeine has been shown to promote an increase in muscle sympathetic nervous activity (34). However, chronic caffeine administration has shown to normalize sympathetic activation and the levels of circulating catecholamines in rats (8), evidencing opposite roles for acute and chronic caffeine consumption.

Due to the contradictory findings regarding the role of adenosine receptors and the beneficial role of chronic caffeine on insulin sensitivity and glucose metabolism, herein, we explored the effect of 15 days administration of DPCPX, SCH58261, and MRS1754, an A_1_, A_2A_, and A_2B_ adenosine receptor antagonists, in a rodent model of insulin resistance. Additionally, we investigated sex differences in the effects of these adenosine receptor antagonists on insulin sensitivity and signaling in insulin-sensitive tissues and on UCP1 expression in the visceral adipose tissue.

## Materials and Methods

### Animals and Experimental Procedures

Experiments were performed in Wistar rats (200–420 g) of both sexes, aged 3 months obtained from the vivarium of the NOVA Medical School|Faculdade de Ciências Médicas of the Universidade Nova de Lisboa, Lisboa, Portugal. Animals were kept under temperature and humidity control (21 ± 1°C; 55 ± 10% humidity) and a regular light (08.00–20.00 h) and dark (20.00–08.00 h) cycle, with food and water *ad libitum*. Animals were assigned to two groups: the control group that fed a standard diet (14.53% protein, 10% fat, 55.06% carbohydrates; RM3, SDS - Special Diet Services, UK) and the high-sucrose diet-fed (HSu) group, that fed the standard diet plus 35% (wt/vol.) sucrose (PanReac, Madrid, Spain) in drinking water for 28 days, representing a lean model of combined insulin resistance and hypertension (35, 36). In the last 15 days of the diet the animals were divided in 3 groups and submitted to the intraperitoneal administration of DPCPX (A_1_ antagonist, 0.4 mg/kg; Sigma, Madrid, Spain), SCH58261 (A_2A_ antagonist, 0.5 mg/kg; Tocris Bioscience, UK), or MRS1754 (A_2B_ antagonist, 9.5 μg/kg; Sigma, Madrid, Spain). All adenosine receptors antagonists were soluble in dimethyl sulfoxide (DMSO), and therefore to take into account the effects of DMSO on insulin sensitivity and glucose metabolism, a group of control and HSu rats was also submitted in the last 15 days of the diet to an intraperitoneal administration of DMSO (Vehicle, dilution 1:3). All test groups included males and females. Body weight, energy and liquid intake were monitored two times per week. At the end of 28 days of diet, animals were tested for insulin sensitivity through an insulin tolerance test (ITT) (8, 36). After the ITT, meaning 15 min post insulin administration, a heart puncture was performed to collect blood, the fat depots were collected and weighted, as well as the insulin sensitive tissues, the liver and skeletal muscle. The tissues were placed on liquid nitrogen and saved at −80°C, until posterior use. Laboratory care was in accordance with the European Union Directive for Protection of Vertebrates Used for Experimental and Other Scientific Ends (2010/63/ EU). Experimental protocols were approved by the NOVA Medical School|Faculdade de Ciências Médicas Ethics Committee.

### Measurement of Insulin Sensitivity

The insulin sensitivity was evaluated by the ITT in animals under sodium pentobarbital (60 mg/kg, i.p.) anesthesia as previously described (8, 36). The ITT consists in the administration of an intravenous insulin (Humulin, 100 IU/ml, Lilly, Lisboa, Portugal) bolus of 0.1 U/kg body weight in the tail vein after an overnight fast, followed by measuring the decline in plasma glucose concentration over 15 min. The constant rate for glucose disappearance (K_ITT_) was calculated using the formula 0.693/t_1/2_. Glucose half-time (t_1/2_) was calculated from the slope of the least square analysis of plasma glucose concentrations during the linear decay phase (8, 37). Blood samples were collected by tail snip tecnique and glucose levels were measured with a glucometer (Precision Xtra Meter, Abbott Diabetes Care, Portugal) and test strips (Abbott Diabetes Care, Portugal).

### Measurement of Plasma Catecholamines Levels and Adenal Medulla Catecholamine Content

To quantify plasma catecholamines, 500 μl of plasma samples were purified and catecholamines were extracted using 30 mg OASIS Hlb Wat cartridges (Waters, Milford, MA, USA) and eluted in 500 μl of mobile phase as previously described (38). For quantification of catecholamine content in adrenal medulla, the organs previously frozen were homogenized in 0.6N perchloric acid, centrifuged at 13,000 g at 4°C and collected the supernatant. One hundred microliters of the samples were directly injected into a high-performance liquid chromatography system composed of a Waters 600 controller pump, a Waters C18 (particle size 4 μm) column, a Waters 717 plus autosampler, a Bioanalytical Systems LC-4A electrochemical detector (set at a holding potential of 0.65 mV and a sensitivity of 1 nA). An isocratic elution was used: the mobile phase consisted of a solution of Na_2_HP_4_ (25 mM) with 6% of methanol (pH 3.55), running at a flux of 1 ml/min. The signal coming out of the detector was fed to an analog to digital converter controlled by Peak Sample Chromatography System Software (Buck Scientific, East Norwalk, CT, USA). Identification and quantification of catecholamines were done against external standards.

### Western Blot Analysis of Adenosine A_1_, A_2A_, and A_2B_ Receptors, Insulin Receptor (IR), Protein Kinase B (Akt), Glucose Transporter Type 4 (GLUT4) or 2 (GLUT2), and Uncoupling Protein 1 (UCP1) in Skeletal Muscle, Liver, and Visceral Adipose Tissue

Skeletal muscle (50 mg), liver (50 mg), and visceral adipose tissue (100 mg) were homogenized in Zurich medium containing a cocktail of protease inhibitors (39). Samples were centrifuged (Eppendorf, Madrid, Spain) at 13,000 g for 20 min and the supernatant was collected and frozen at −80°C until further use. The evaluation of adenosine receptors A_1_, A_2A_, and A_2B_, UCP1, GLUT4, GLUT2, insulin receptor, insulin receptor phosphorylated at Tyr1361, Akt, and Akt phosphorylated at Ser473 was performed according to Sacramento et al. (39) and Matafome et al. (40). Briefly, after blocking for 1 h at room temperature with 5% non-fat milk in Tris-buffered saline (TBS), pH 7.4 containing 0.1% Tween 20 (TTBS) (BioRad, Spain), the membranes were incubated overnight at 4°C with the primary antibodies against A_1_ (1:200), A_2A_ (1:200), A_2B_ (1:200), GLUT4 (1:200), GLUT2 (1:200), insulin receptor (1:200) (Sta Cruz Biotechnology, USA), insulin receptor phosphorylated (phospho-Tyr1361, 1:500; Abcam, UK), Akt (1:1,000, Cell Signaling, USA), Akt phophorylated (phospho-Ser473, 1:1,000, Cell Signaling, USA), and UCP1 (1:1,000, Abcam, USA). The membranes were washed with Tris-buffered saline with 0.1% TBST and incubated with donkey anti-goat (1:2,000, Sta Cruz Biotechnology, USA) or goat anti-mouse (1:2,000, Sta Cruz Biotechnology, USA) or goat anti-rabbit (1:5,000, Rockland, USA) in TTBS for 2 h at room temperature and developed with enhanced chemiluminescence reagents according to the manufacturer's instructions (ClarityTM Western ECL substrate, BioRad, United States). Intensity of the signals was detected in a Chemidoc Molecular Imager (Chemidoc; BioRad, Madrid, Spain) and quantified using the Quantity-One software (BioRad, Madrid). We tested the expression of UCP1 in brown adipose tissue (BAT) as positive controls. The membranes were re-probed and tested for Calnexin (1:1,000, SicGen, Portugal), α-Tubulin (1:1,000, Sta Cruz Biotechnology, USA), or GAPDH (1:250, Sta Cruz Biotechnology, USA) immunoreactivity (bands in the 90, 55, and 37 kDa region, respectively) to compare and normalize the expression of proteins with the amount of protein loaded. Different loading proteins were used in accordance with the molecular weight of the protein to be studied or with the tissue in where protein expression was analyzed.

### Data Analysis

Data were analyzed using GraphPad Prism Software, version 6 (GraphPad Software Inc., San Diego, CA, EUA) and were presented as mean ± SD. The significance of the differences between the means was calculated by One and Two-Way Analysis of Variance (ANOVA) with Dunnett's and Bonferroni multicomparison test, respectively. *p-*values of 0.05 or less were considered to represent significant differences.

## Results

Liquid intake (milliliters/day) was similar in all groups of animals tested and the administration of the vehicle, DPCPX, SCH58261, and MRS1754 during 15 days did not modify liquid intake or animal behavior within groups (data not shown).

### Effect of Chronic Administration of A_1_, A_2A_, and A_2B_ Adenosine Receptor Antagonists on Insulin Sensitivity and Fasting Glycaemia

**Figure 1:**
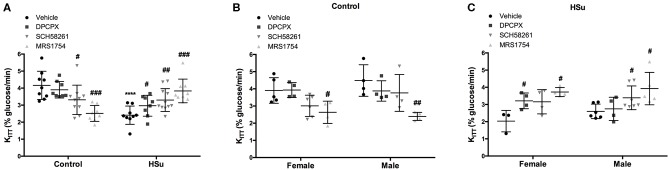
Effect of chronic administration of A_1_, A_2A_, and A_2B_ adenosine receptor antagonists on insulin sensitivity in control and high-sucrose (HSu) animals. **(A)** Insulin sensitivity in animals of both sexes; **(B,C)** Gender differences in insulin sensitivity in control and HSu animals, respectively. Insulin sensitivity was determined by the insulin tolerance test (ITT) and expressed as constant for glucose disappearance (K_ITT_). Vehicle (DMSO, dilution 1:3), DPCPX (A_1_ antagonist, 0.4 mg/kg), SCH58261 (A_2A_ antagonist, 0.5 mg/kg), and MRS1754 (A_2B_ antagonist, 9.5 μg/kg) were administered i.p. during 15 days. Values represent mean±SD of 8–11 animals of both sexes. One- and Two-Way ANOVA with Dunnett's and Bonferroni multicomparison tests, respectively: ****p < 0.0001 vs. vehicle (control); #*p* < 0.05, ##*p* < 0.01 and ###*p* < 0.001 comparing values with vehicle in the same group.

Figure 1 depicts the effect of chronic adenosine receptor antagonist administration on insulin sensitivity in control and HSu animals. Chronic blockade of A_2A_ adenosine receptor by SCH58261 and A_2B_ adenosine receptor by MRS1754 decreased significantly insulin sensitivity in control animals from a control value of 4.16 ± 0.83 to 3.31 ± 0.91% glucose/min and to 2.52 ± 0.47% glucose/min, respectively (Figure 1A). Administration of DPCPX, a selective A_1_ adenosine receptor antagonist, in control animals was unable to change insulin sensitivity (Figure 1A). As previously described by Conde et al. (8), ingestion of HSu diet during 28 days induced insulin resistance (K_ITT_ HSu = 2.41 ± 0.54% glucose/min) (Figure 1A). Chronic administration of DPCPX and SCH58261 improved insulin sensitivity by 23.24 and 36.93%, respectively (Figure 1A) in HSu animals. Moreover, blockade of A_2B_ receptors almost restored insulin sensitivity induced by HSu diet (K_ITT_ HSu+MRS1754 = 3.84 ± 0.70% glucose/min). These effects of adenosine antagonists on insulin sensitivity in control and HSu groups followed the same pattern if animals were separated by sexes: females and males (Figures 1B,C). In control animals, chronic blockade of A_1_ receptors increased significantly by 16.25% fasting glycaemia (Table 1), whereas the blockade of A_2A_ and A_2B_ receptors did not produced any alteration. Chronic administration of HSu diet or of the different adenosine receptor antagonists did not modify significantly fasting glycaemia in HSu animals (Table 1).

**Table 1:** Effect of chronic A_1_, A_2A_, and A_2B_ adenosine receptor antagonist administration on fasting glycemia, in male and female rats submitted to a standard diet and to a high sucrose (HSu) diet.

**Treatment**		**Vehicle**	**DPCPX**	**SCH58261**	**MRS1754**
Control	All animals	86.66 ± 10.90	100.75 ± 10.05^*^	82.29 ± 4.39	84.13 ± 4.61
	Females	87.00 ± 12.35	102.75 ± 10.90	82.00 ± 4.56	83.25 ± 4.19
	Males	86.25 ± 10.63	99.50 ± 10.72	82.50 ± 4.57	85.00 ± 5.48
HSu	All animals	100.00 ± 20.82	108.25 ± 7.76	91.64 ± 9.87	93.77 ± 10.27
	Females	105.00 ± 35.66	105.50 ± 7.04	89.75 ± 9.98	97.25 ± 9.43
	Males	97.50 ± 13.81	111.00 ± 8.40	92.71 ± 10.44	91.00 ± 11.09

Values represent mean±SD of 8–11 animals of both sexes. Vehicle (DMSO, dilution 1:3); DPCPX (A_1_ antagonist, 0.4 mg/kg), SCH58261 (A_2A_ antagonist, 0.5 mg/kg), and MRS1754 (A_2B_ antagonist, 9.5 μg/kg). One- and Two-Way ANOVA with Dunnett's and Bonferroni multicomparison tests, respectively:

* *p < 0.05 vs. vehicle*.

### Effect of Chronic Administration of A_1_, A_2A_, and A_2B_ Adenosine Receptor Antagonists on Weight Gain and Fat Depots

**Figure 2:**
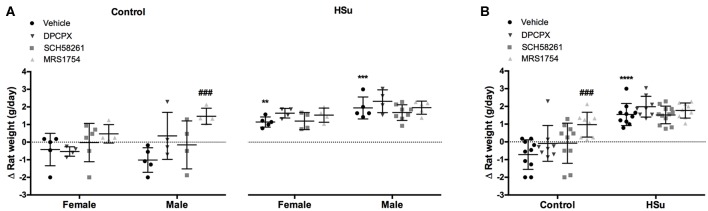
Effect of chronic A_1_, A_2A_ and A_2B_ adenosine receptor antagonist administration on body-weight increment, calculated as total weight variation during the experiment period. **(A)** Gender differences in weight increment in control and HSu female and male animals. **(B)** Weight gain in animals of both sexes. Vehicle (DMSO, dilution 1:3), DPCPX (A_1_ antagonist, 0.4 mg/kg), SCH58261 (A_2A_ antagonist, 0.5 mg/kg), and MRS1754 (A_2B_ antagonist, 9.5 μg/kg) were administered i.p. during 15 days. Values represent mean±SD of 8–12 animals of both sexes. One- and Two-Way ANOVA with Dunnett's and Bonferroni multicomparison tests, respectively: **p < 0.01, ***p < 0.001 and ****p < 0.0001 vs. vehicle (control); ###*p* < 0.001 comparing values with vehicle in the same group.

HSu diet promoted an increase in weight gain (Figure 2), being the increase higher in males than in females (females = 2.27 ± 0.55 g/day; males = 3.96 ± 1.21 g/day). None of the adenosine antagonists tested altered weight gain in control or HSu animals, except the A_2B_ antagonist, that increased by 211.90 and 244.19% weight gain in control female and male, respectively (Figure 2A).

The effect of chronic administration of adenosine receptor antagonists on total, perienteric/visceral, genital, and perinephric fat in control and HSu males and females is shown in Table 2. Male control rats exhibit a higher total fat content than control females (Table 2). Administration of A_1_ and A_2B_ blockers did not modify significantly nor the total fat amount neither the deposition of fat in the distinct fat depots, genital, perienteric, or perinephric in control males or females. Chronic administration of A_2A_ blocker increased by 50.39% and decreased by 18.41% total fat amount, in control female and male animals, respectively (Table 2). These effects might be due to the 111.98% increase in the genital fat of female rats and to the 27.80% decrease in the perienephric fat of control males (Table 2). Interestingly, female HSu rats exhibit a higher total fat content than HSu males (Table 2). In HSu females, chronic A_1_ blockade decreased by 29.61, 22.10, 24.38, and 43.55% the total, perienteric, genital, and perinephric fat, respectively, while A_2B_ blockade increased by 20.75% the total fat (Table 2). In male HSu animals, chronic administration of A_2A_ and A_2B_ antagonists increased by 24.19 and 20.10% the total fat, respectively, and by 25.32 and 22.59% the perinephric fat, respectively (Table 2). Concluding, male control rats exhibit higher total fat content than females, in contrast to what happens in HSu diet in which females exhibit more fat content than males. A_1_ receptor blockade in insulin resistant states lead to fat loss in female rats while the blockade of A_2_ receptors lead to an increase in total fat both in males and females.

**Table 2:** Effect of chronic A_1_, A_2A_, and A_2B_ adenosine receptor antagonist administration on total, visceral/perienteric, genital, and perinephric fat in control and high-sucrose (HSu) female and male animals.

**Treatment**			**Vehicle**	**DPCPX**	**SCH58261**	**MRS1754**
Female	CTL	Total	31.83 ± 8.93	25.78 ± 9.47	47.87 ± 9.59^##^	49.59 ± 33.22
		Perienteric	7.75 ± 3.15	4.63 ± 2.33	8.12 ± 1.78	26.48 ± 22.27
		Genital	12.85 ± 1.72	13.92 ± 3.09	27.24 ± 6.92^##^	48.13 ± 11.50
		Perinephric	11.23 ± 6.03	7.22 ± 5.50	12.51 ± 1.64	13.26 ± 4.64
	HSu	Total	72.71 ± 13.85^***^^,^ ^§§^	50.34 ± 7.47^###^	83.68 ± 8.45	87.80 ± 5.65^#^
		Perienteric	9.57 ± 2.46	6.74 ± 1.56	13.46 ± 1.32	12.44 ± 1.57
		Genital	37.15 ± 7.75	28.94 ± 4.19	44.82 ± 8.56	45.98 ± 4.11
		Perinephric	25.99 ± 5.29	14.67 ± 2.98^##^	25.40 ± 1.44	29.38 ± 3.72
Male	CTL	Total	44.27 ± 1.18^§^	46.71 ± 4.81	36.12 ± 1.72^##^	47.43 ± 4.03
		Perienteric	6.59 ± 0.23	7.22 ± 0.89	6.06 ± 0.25	7.17 ± 0.30
		Genital	16.96 ± 0.69	18.37 ± 2.27	15.08 ± 0.59	19.00 ± 1.15
		Perinephric	20.72 ± 1.29	21.11 ± 2.05	14.96 ± 1.07^#^	21.26 ± 1.15
	HSu	Total	51.64 ± 5.92	47.02 ± 1.32	64.13 ± 5.51^####^	62.02 ± 8.25^###^
		Perienteric	8.49 ± 1.06	7.82 ± 0.68	9.95 ± 1.49	8.40 ± 1.36
		Genital	18.98 ± 2.11	16.57 ± 2.98	23.88 ± 1.84	21.87 ± 3.69
		Perinephric	24.17 ± 3.21	22.63 ± 1.96	30.29 ± 3.66^#^	29.63 ± 3.01^#^

Values represent mean±SD of 4–8 animals. Vehicle (DMSO, dilution 1:3); DPCPX (A_1_ antagonist, 0.4 mg/kg), SCH58261 (A_2A_ antagonist, 0.5 mg/kg), and MRS1754 (A_2B_ antagonist, 9.5 μg/kg). One- and Two-Way ANOVA with Dunnett's and Bonferroni multicomparison tests, respectively:

*** p < 0.001 vs. control values in the same sex;

# p < 0.05,

## p < 0.01,

### p < 0.001, and

#### p < 0.0001 comparing values with vehicle in the same group;

§ p < 0.05;

§§ *p < 0.01 comparing female with male animals*.

### Effect of Chronic Administration of A_1_, A_2A_, and A_2B_ Adenosine Receptor Antagonists on Plasma and Adrenal Medulla Catecholamines

To evaluate the effect of chronic administration of A_1_, A_2A_, and A_2B_ adenosine receptor antagonists on sympathetic nervous system activity, we measured both circulating and adrenal medulla catecholamines content in control and HSu animals (Table 3 and Figure 3). Values are presented in Table 3 separated by sexes and in Figure 3 plotted together. As expected and previously described (36) HSu diet increased plasma catecholamines and adrenal medulla Epi content (Figures 3A–C). Chronic administration of adenosine receptor antagonists did not modify circulating NE and Epi in both control and HSu animals (Figures 3A,B). Control animals submitted to chronic A_1_ and A_2B_ adenosine receptor blockade exhibited significant increases of 252.07 and 209.68% in adrenal medulla NE, respectively, and of 172.22 and 128.30% in adrenal medulla Epi content, respectively, compared with the control animals (NE control vehicle = 2.17 ± 0.78 nmol/mg tissue; Epi control vehicle = 9.54 ± 2.76 nmol/mg tissue) (Figures 3C,D). Chronic A_1_ and A_2B_ adenosine receptor blockade also increased significantly the adrenal medulla NE and Epi content in HSu animals (Figures 3C,D). SCH58261 chronic administration induced an increase of 37.52% in adrenal medulla Epi content in control animals (Figure 3D) and of 47.47% in adrenal medulla NE content in HSu animals (Figure 3C).

**Table 3:** Effect of chronic A_1_, A_2A_, and A_2B_ adenosine receptor antagonist administration on circulating and adrenal medulla catecholamines, norepinephrine and epinephrine, in male and female rats submitted to a standard diet and to a high sucrose diet.

**Treatment**				**Vehicle**	**DPCPX**	**SCH58261**	**MRS1754**
Plasma	CTL	NE	Females	20.74 ± 16.99	43.15 ± 15.77	10.72 ± 4.80	49.59 ± 33.22
			Males	13.63 ± 13.64	9.7 ± 7.65	17.76 ± 9.76	26.48 ± 22.27
		Epi	Females	53.69 ± 28.38	60.61 ± 17.83	48.63 ± 22.21	48.13 ± 11.50
			Males	40.50 ± 27.70	26.46 ± 21.15	34.33 ± 18.95	55.97 ± 20.82
	HSu	NE	Females	36.32 ± 0.36	46.21 ± 34.58	22.19 ± 21.98	32.47 ± 29.13
			Males	38.49 ± 11.48	98.41 ± 26.49	14.42 ± 11.83	45.90 ± 25.10
		Epi	Females	84.73 ± 24.87	63.52 ± 35.71	42.57 ± 7.15	52.49 ± 32.29
			Males	44.36 ± 42.42	72.93 ± 9.39	28.64 ± 19.39	69.56 ± 21.30
Adrenal medulla	CTL	NE	Females	1.89 ± 0.82	6.30 ± 4.18	3.25 ± 1.74	5.27 ± 2.98
			Males	2.45 ± 0.74	9.00 ± 3.12^##^	3.05 ± 0.46	8.65 ± 2.10^#^
		Epi	Females	9.30 ± 3.47	22.12 ± 2.85^##^	14.03 ± 3.48	18.80 ± 9.43^#^
			Males	9.79 ± 2.35	29.81 ± 0.88^####^	10.38 ± 2.03	25.75 ± 3.68^###^
	HSu	NE	Females	1.70 ± 0.95	5.53 ± 1.60^###^	3.97 ± 1.02^#^	5.06 ± 1.49^##^
			Males	2.87 ± 1.34	6.65 ± 0.44^##^	3.54 ± 0.76	8.30 ± 1.23^####^
		Epi	Females	11.92 ± 7.07	21.95 ± 5.80^###^	12.10 ± 0.14^#^	18.84 ± 6.07^##^
			Males	12.92 ± 3.92	24.54 ± 9.63	16.26 ± 2.93	25.72 ± 5.63^##^

Values represent mean±SD of 4–8 animals. Vehicle (DMSO, dilution 1:3); DPCPX (A_1_ antagonist, 0.4 mg/kg), SCH58261 (A_2A_ antagonist, 0.5 mg/kg), and MRS1754 (A_2B_ antagonist, 9.5 μg/kg). Two-Way ANOVA with Bonferroni multicomparison tests, respectively:

# p < 0.05,

## p < 0.01,

### p < 0.001, and

#### *p < 0.0001 comparing values with vehicle in the same group*.

**Figure 3:**
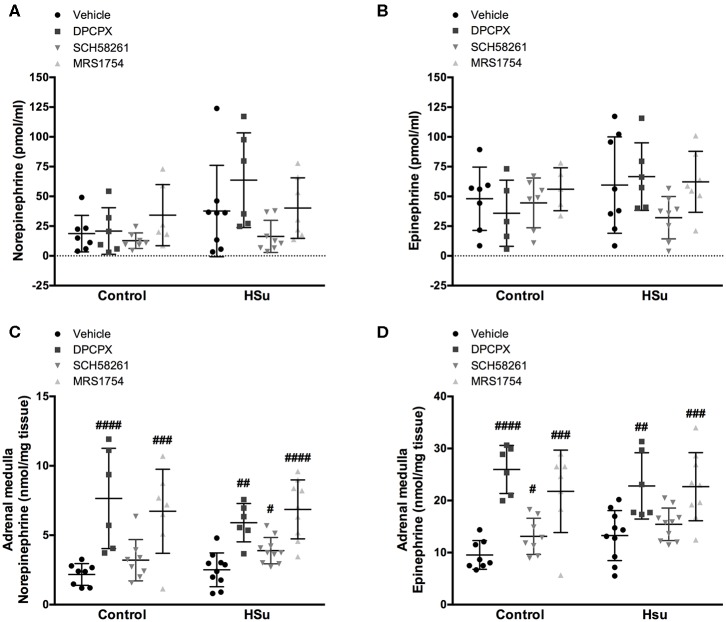
Effect of chronic A_1_, A_2A_, and A_2B_ adenosine receptor antagonist administration on circulating catecholamines, norepinephrine and epinephrine, and on adrenal medulla norepinephrine and epinephrine content. **(A,B)** Shows respectively the impact of 15 days of A_1_, A_2A_, and A_2B_ adenosine receptor antagonist administration on circulating catecholamines, norepinephrine, and epinephrine. **(C,D)** Shows respectively the impact of 15 days of A_1_, A_2A_, and A_2B_ adenosine receptor antagonist administration on adrenal medulla norepinephrine and epinephrine content. Vehicle (DMSO, dilution 1:3), DPCPX (A_1_ antagonist, 0.4 mg/kg), SCH58261 (A_2A_ antagonist, 0.5 mg/kg), and MRS1754 (A_2B_ antagonist, 9.5 μg/kg) were administrated i.p. during 15 days. Values represent mean±SD of 6–11 animals. One- and Two-Way ANOVA with Dunnett's and Bonferroni multicomparison tests, respectively: #*p* < 0.05, ##*p* < 0.01, ###*p* < 0.001, ####*p* < 0.0001 comparing values with vehicle in the same group.

### Effect of Chronic Administration of A_1_, A_2A_, and A_2B_ Adenosine Receptor Antagonists on A_1_, A_2A_, and A_2B_ Expression

**Figure 4:**
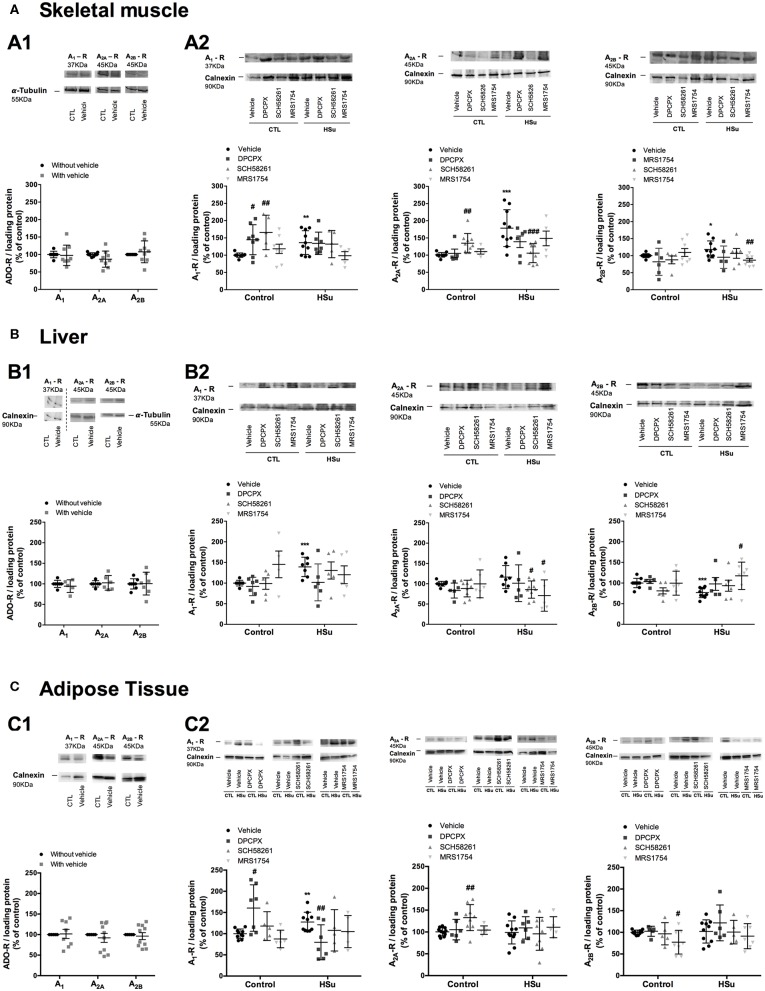
Effect of chronic A_1_, A_2A_, and A_2B_ adenosine receptor antagonist administration on the expression of its receptors on the insulin sensitivity tissues, skeletal muscle, liver, and visceral fat. **(A1,B1,C1)** Shows respectively the impact of vehicle administration on the expression of the adenosine tested in the skeletal muscle, liver, and visceral fat of control animals. **(A2,B2,C2)** Shows respectively the impact of 15 days of A_1_, A_2A_, and A_2B_ adenosine receptor antagonist administration on adenosine receptor expression in the skeletal muscle, liver, and visceral fat of control and HSu animals in relation to the expression of the loading protein. Vehicle (DMSO, dilution 1:3), DPCPX (A_1_ antagonist, 0.4 mg/kg), SCH58261 (A_2A_ antagonist, 0.5 mg/kg), and MRS1754 (A_2B_ antagonist, 9.5 μg/kg) were administrated i.p. during 15 days. Values represent mean±SD of 5–10 animals. One- and Two-Way ANOVA with Dunnett's and Bonferroni multicomparison tests, respectively: *p < 0.05, **p < 0.01, and ***p < 0.001, vs. vehicle (control); #*p* < 0.05, ##*p* < 0.01, and ###*p* < 0.001 comparing values with vehicle in the same group.

No significant changes were observed for the effect of chronic DPCPX, SCH58261, and MRS1754 administration on the expression of A_1_, A_2A_, and A_2B_ in insulin sensitive tissues expressed by sex (data not shown), thereby the results of female and male animals were plotted together (Figure 4). DMSO can interfere with various cellular processes (41), but herein DMSO did not modify the expression of A_1_, A_2A_, and A_2B_ in skeletal muscle, liver, and adipose tissue (Figures 4A1,B1,C1). Chronic DPCPX and SCH58261 increased significantly by 44.72 and 65.82% the expression of A_1_, respectively, in the skeletal muscle from control animals (Figure 4A2). Additionally, chronic SCH58261 administration increased by 34.85% the expression of A_2A_ receptors, but MRS1754 did not alter the expression of the different adenosine receptors in skeletal muscle (Figure 4A2). HSu diet itself caused a significant increase of 78.57 and 18.18% in the expression of A_2A_ and A_2B_ adenosine receptors, respectively, an effect that was restored by the chronic administration of SCH58261 and MRS1754, respectively (Figure 4A2). The expression of A_1_ adenosine receptors was also increased by 36.33% with the HSu diet, but chronic DPCPX administration did not altered A_1_ receptor expression in skeletal muscle (Figure 4A2).

In the liver, chronic administration of DPCPX, SCH58261, and MRS1754 in control animals did not modify the expression of A_1_, A_2A_, and A_2B_ adenosine receptors, respectively (Figure 4B2). HSu diet increased by 73.64 and 16.47% (*p* = 0.091) the expression of A_1_ and A_2A_ adenosine receptors, respectively (Figure 4B2), but decreased by 23.17% the expression of adenosine A_2B_ receptors, being this last effect restored by chronic MRS1754 administration (Figure 4B2).

Following the same profile than the skeletal muscle, in the adipose tissue from control animals, chronic administration of DPCPX and SCH58261 increased by 60.44 and 37.54% the expression of A_1_ and A_2A_ adenosine receptors, respectively. Although in this tissue the administration of MRS1754 decreased by 22.80% the expression of A_2B_ adenosine receptors (Figure 4C2). HSu diet or the administration of DPCPX, SCH58261, and MRS1754 in this insulin-resistant animal model did not modify the expression of A_1_, A_2A_, and A_2B_ receptors in adipose tissue (Figure 4C2). Generally and as expected, the selective blockade of the different adenosine receptors produced an upregulation of these receptors in insulin sensitive tissues. HSu diet increased A_1_, A_2A_, and A_2B_ receptor expression in the skeletal muscle, increased A_1_ receptor expression and decreased A_2B_ in the liver, and increased A_1_ in the adipose tissue, effects that were rescued by the blockade of the respective adenosine receptors.

### Effect of Chronic A_1_, A_2A_, and A_2B_ Adenosine Receptor Antagonist Administration on Insulin Signaling Pathways

**Figure 5:**
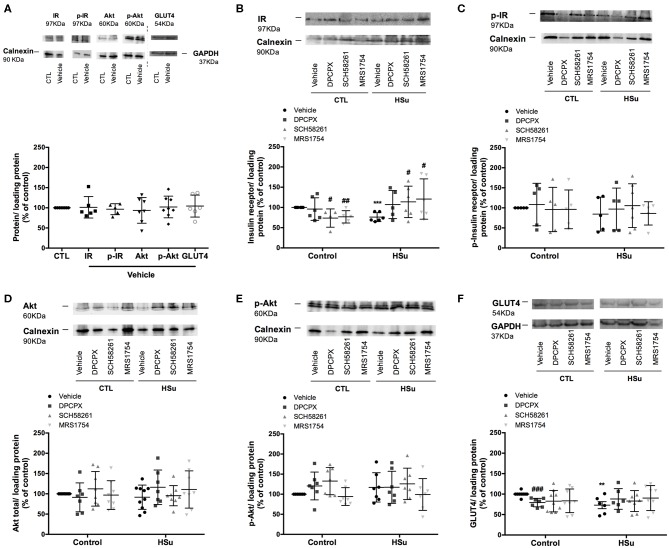
Effect of chronic A_1_, A_2A_, and A_2B_ adenosine receptor antagonist administration on insulin signaling pathways in the skeletal muscle. **(A)** Effect of the vehicle, DMSO, on insulin signaling pathways in control animals. Average relative **(B)** insulin receptor levels (97 kDa band), **(C)** insulin receptor phosphorylation (97 kDa band), **(D)** Akt levels (60 kDa band), **(E)** Akt phosphorylation (60 kDa band), and **(F)** GLUT4 (54 kDa band) immunoreactivity in skeletal muscle from control and HSu animals with or without chronic A_1_, A_2A_, and A_2B_ adenosine receptor antagonist administration in relation to the expression of the loading protein. Representative western blots for each protein studied are depicted above the respective graphs. Vehicle (DMSO, dilution 1:3), DPCPX (A_1_ antagonist, 0.4 mg/kg), SCH58261 (A_2A_ antagonist, 0.5 mg/kg), and MRS1754 (A_2B_ antagonist, 9.5 μg/kg) were administrated i.p. during 15 days. Values represent mean±SD of 5–10 animals. One- and Two-Way ANOVA with Dunnett's and Bonferroni multicomparison tests, respectively: **p < 0.01 and ***p < 0.001 vs. vehicle (control); #*p* < 0.05, ##*p* < 0.01, and ###*p* < 0.001 comparing values with vehicle in the same group.

**Figure 6:**
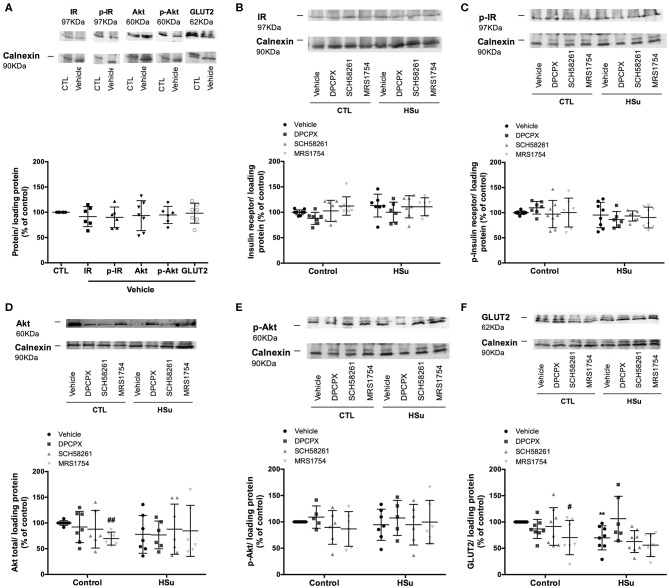
Effect of chronic A_1_, A_2A_, and A_2B_ adenosine receptor antagonist administration on insulin signaling pathways in the liver. **(A)** Effect of the vehicle, DMSO, on insulin signaling pathways in control animals. Average relative **(B)** insulin receptor levels (97 kDa band), **(C)** insulin receptor phosphorylation (97 kDa band), **(D)** Akt levels (60 kDa band), **(E)** Akt phosphorylation (60 kDa band), and **(F)** GLUT2 (62 kDa band) immunoreactivity in the liver from control and HSu animals with or without chronic A_1_, A_2A_, and A_2B_ adenosine receptor antagonist administration in relation to the expression of the loading protein. Representative western blots for each protein studied are depicted above the respective graphs. Vehicle (DMSO, dilution 1:3), DPCPX (A_1_ antagonist, 0.4 mg/kg), SCH58261 (A_2A_ antagonist, 0.5 mg/kg), and MRS1754 (A_2B_ antagonist, 9.5 μg/kg) were administrated i.p. during 15 days. Values represent mean±SD of 5–10 animals. One- and Two-Way ANOVA with Dunnett's and Bonferroni multicomparison tests, respectively: **p < 0.01 vs. vehicle (control); #*p* < 0.05 and ##*p* < 0.01 comparing values with vehicle in the same group.

**Figure 7:**
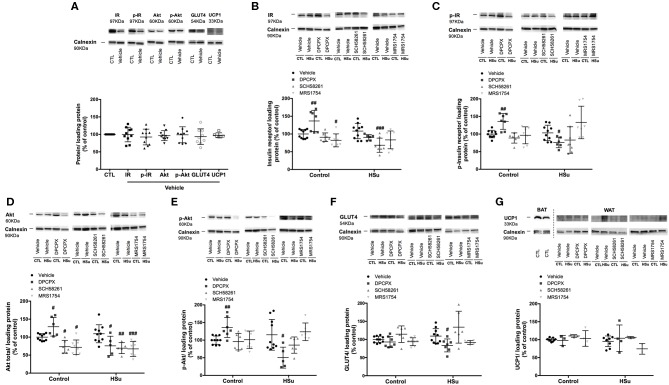
Effect of chronic A_1_, A_2A_, and A_2B_ adenosine receptor antagonist administration on insulin signaling pathway and UCP1 expression in the visceral/perienteric adipose tissue. **(A)** Effect of the vehicle, DMSO, on insulin signaling pathways in control animals. Average relative **(B)** insulin receptor levels (97 kDa band), **(C)** insulin receptor phosphorylation (97 kDa band), **(D)** Akt levels (60 kDa band), **(E)** Akt phosphorylation (60 kDa band), **(F)** GLUT4 (54 kDa band), and **(G)** UCP1 (33 kDa band) immunoreactivity in white visceral adipose tissue (WAT) from control and HSu animals with or without chronic A_1_, A_2A_, and A_2B_ adenosine receptor antagonist administration in relation to the expression of the loading protein. Representative western blots for each protein studied are depicted above the respective graphs. A positive control for UCP1 in brown adipose tissue (BAT) was used **(G)**. Vehicle (DMSO, dilution 1:3), DPCPX (A_1_ antagonist, 0.4 mg/kg), SCH58261 (A_2A_ antagonist, 0.5 mg/kg), and MRS1754 (A_2B_ antagonist, 9.5 μg/kg) were administrated i.p. during 15 days. Values represent mean±SD of 5–10 animals. One- and Two-Way ANOVA with Dunnett's and Bonferroni multicomparison tests, respectively: #*p* < 0.05, ##*p* < 0.01, and ###*p* < 0.001, comparing values with vehicle in the same group.

No sex differences were seen for effect of chronic DPCPX, SCH58261, and MRS1754 administration on insulin signaling pathways, and therefore results were expressed together (Figures 5–7). DMSO, the vehicle used in this study, did not modify the levels and activity of insulin receptor, Akt and GLUT4 levels in the skeletal muscle, liver, and adipose tissue (Figures 5–7A). In the skeletal muscle from control animals, chronic administration of SCH58261 and MRS1754 decreased significantly by 26.27 and 23.21% insulin receptor levels (Figure 5B). Insulin receptor phosphorylation, Akt levels and phosphorylation did not change with chronic administration of the different adenosine receptor antagonists in control animals (Figures 5C–E). GLUT4 levels in control animals decreased by 20.68, 17.29, and 16.40% with chronic DPCPX, SCH58261, and MRS1754 administration, respectively (Figure 5F). As expected and consistent with the development of insulin resistance, HSu diet decreased insulin receptor expression and GLUT4 levels by 23.89 and by 27.00%, respectively, without any effect on insulin receptor and Akt phosphorylation. In HSu animals, chronic administration of DPCPX, SCH58261, and MRS1754 increased by 40.81 (*p* = 0.066), 49.52 and 58.68% insulin receptor levels (Figure 5B). Chronic administration of the different adenosine receptor antagonists did not modify insulin receptor and Akt phosphorylation and GLUT4 levels (Figures 5C,E,F).

In the liver in control animals, chronic administration of DPCPX and SCH58261 did not modify insulin receptor, Akt levels and their phosphorylation, whereas MRS1754 administration decreased by 30.31 and 29.66% Akt and GLUT2 levels, respectively (Figures 6B–F). HSu diet ingestion during 4 weeks did not alter levels and phosphorylation of insulin receptor and Akt, while decreased GLUT2 levels by 52.27% in liver, an effect restored with the chronic administration of DPCPX (*p* = 0.060) (Figures 6B–F).

In the visceral/perienteric adipose tissue, chronic administration of DPCPX in control animals increased insulin receptor levels and its phosphorylation, Akt levels and its phosphorylation by 36.81, 36.00, 28.86, and 36.10%, respectively, without altering Glut4 levels (Figures 7B–F). In contrast, chronic MRS1754 administration decreased by 17.92 and 28.28% insulin receptor values and Akt levels, respectively (Figures 7B,D). Moreover, chronic SCH58261 administration decreased by 26.60% Akt levels (Figure 7D). HSu diet *per se* was unable to alter the levels and activity of insulin receptor, Akt and GLUT4 levels (Figures 7B–F). In HSu animals, insulin receptor levels and its phosphorylation decreased by 36.91 and 26.31% with chronic administration of SCH58261 and DPCPX, respectively (Figures 7B,C). Chronic DPCPX and SCH58261 administration decreased by 30.71 and 36.55% Akt levels and by 64.83 and 33.48% Akt phosphorylation, respectively, although this last effect of SCH58261 on Akt phosphorylation was non–significant (Figures 7D,E). MRS1754 only decreased Akt values by 38.43% (Figures 7D,E). Also, in the adipose tissue from HSu animals, chronic DPCPX administration decreased by 23.56% GLUT4 levels (Figure 7F).

### Effect of Selective Adenosine Receptor Blockade on UCP1 Expression in the Visceral Adipose Tissue

One of the mechanisms described to be associated with the beneficial caffeine consumption on metabolism is the increase in thermogenesis (29). The innervation of the brown adipose tissue by the sympathetic nervous system function as the principal stimulator of brown adipose tissue thermogenesis, being the activation of the sympathetic nervous system known also as one of the main triggers for the browning/beiging of the white adipose tissue (42). The beiging of the visceral white adipose tissue was evaluated as the expression of UCP1. From the observation of Figure 7G in the representative western blots and as expected, it is clear that the levels of UCP1 expression in the visceral adipose tissue are quite small in comparison with the levels of expression in brown adipose tissue. Also, HSu diet did not modify UCP1 expression in the visceral adipose tissue (*p* = 0.400) as well as none of the adenosine receptor antagonists changed the expression of UCP1 (Figure 7G).

## Discussion

In the present study, we demonstrated that adenosine receptors exhibit opposite effects on insulin sensitivity, as chronic adenosine antagonists in control animals promote insulin resistance meaning that adenosine is an insulin-sensitizer and that in insulin-resistant conditions, in HSu animals, chronic antagonists rescue the insulin-resistance phenotype. Additionally, we showed that the role of adenosine receptors in whole-body insulin sensitivity is manly mediated by adenosine A_2_ receptors, with a contribution from adenosine A_1_ receptors, an effect similar between female and male animals. Consistent with these findings, A_2A_ and A_2B_ adenosine receptors antagonists rescued impaired insulin signaling pathways in the skeletal muscle. In contrast, the antagonists tested herein did not alter significantly insulin signaling in the liver, except the A_2B_ antagonist that decreased Akt and GLUT2 expression in control animals, and contribute for the deregulation of insulin signaling pathways in the adipose tissue in HSu animals. In agreement with the improvement of insulin signaling pathways in the skeletal muscle of HSu animals, these animals showed increased expression of A_2A_ and A_2B_ receptor expression that was restored with the chronic blocking of these receptors.

We also show that the effect of chronic adenosine blockade on fat depots was different between females and males, with fat depots accumulation being decreased in female HSu animals submitted to the inhibition of A_1_ adenosine receptors, while increased with the inhibition of A_2A_ and A_2B_ adenosine receptors in male HSu animals. HSu hypercaloric diet promoted the overexpression of A_1_ receptors in the adipose tissue, an, effect rescued by A_1_ receptors blockade. Chronic A_1_ and A_2B_ adenosine blocking induced an increase in sympatho-adrenal activity that did not correlate with the activation of the thermogenesis in the white adipose tissue.

All together, the results herein described strongly suggest that A_2_ adenosine receptors in the skeletal muscle and A_1_ receptors in the adipose tissue are majors contributors for the whole-body insulin sensitivity and that in the context of lean insulin resistance, as the HSu hypercaloric diet, the effect of adenosine on insulin action on skeletal muscle is more relevant than the effect of adenosine on adipose tissue.

### Role of Adenosine Receptors on Whole-Body Insulin Sensitivity and Action

The present study was focused on adenosine A_1_, A_2A_, and A_2B_ receptors, since the role of A_3_ adenosine receptors on insulin sensitivity and glucose homeostasis is not completely elucidated (43). Paradoxically, while in control animals, chronic blockade of A_2A_ and A_2B_ receptor induced insulin resistance in HSu animals, a model of insulin resistance, administration of these antagonists almost prevented insulin resistance, an effect similar between females and males. These results suggest that the beneficial effects of chronic caffeine consumption, a non-selective adenosine receptor antagonist, in lowering the risk of develop type 2 diabetes (5–7), might be mediated by adenosine A_2_ receptors, with a minor contribution from A_1_ adenosine receptors.

In control animals, chronic blockade of A_1_ receptor, showed a tendency to increase fasting glycaemia, which might suggest that in control animals, adenosine acting on A_1_ receptors might contribute to improve glucose metabolism. This is in agreement with the findings of Faulhaber-Walter et al. (11) evidencing that A_1_ receptor deletion impair glucose metabolism and with our results that show that blockade of A_1_ receptors worse insulin signaling dysfunction in the adipose tissue in HSu animals (Figure 7), but differ from the increase in insulin signaling pathways in control animals (Figure 7), reflecting once more the opposite effects in control vs. disease conditions. In contrast, blockade of A_2A_ and A_2B_ receptors showed a tendency to decrease the heightened levels of glucose induced by the HSu diet, which may be an indirect consequence of improved peripheral insulin sensitivity (Figure 1). We can conclude that adenosine receptors exert opposite actions in modulating insulin action in control and insulin resistant animals, with A_2_ chronic adenosine receptors antagonists promoting insulin resistance in control animals and rescuing this phenotype in insulin-resistance states.

### Role of Adenosine Receptors in Weight, Fat Deposition, and Metabolism

The effect of adenosine and its receptors in weight and body fat deposition are not consensual, with some studies showing that the loss of adenosine A_1_ and A_2B_ receptors promote an increase in weight gain and fat deposition (11, 44) and that long-term caffeine consumption is associated with weight loss in rodents and humans (45), effects that are not observed in other studies [e.g., Astrup et al. (29)]. Here, we show that in control animals, chronic blockade of A_2B_ receptors increased weight gain with a higher impact in male animals (Figure 2), an effect that cannot be attributed to an increase in fat mass (Table 2). This effect is in agreement with the study by Csóka et al. (44), in where mice lacking A_2B_ receptors increased weight gain, however due to an increase in retroperitoneal and epididymal fat mass. Surprisingly, chronic A_2A_ blockade, increased total fat in female, while decreased total fat mass in male, which contrasts with the lack of effects of the deletion of A_2A_ receptors in mice in body weight (46). As previously described, 4 weeks of HSu diet were unable to promote significant changes in weight gain (8, 47), being this effect also observed with the blockade of the adenosine receptors. However, even without any change in weight gain, chronic blockade of A_1_ receptors in HSu female animals decreased total fat mass, which was associated with a decrease in perienteric, genital, and perinephric fat mass, suggesting a redistribution of fat depots or an altered ratio lean/fat mass. This decrease in fat mass mediated by A_1_ adenosine receptors may be due to an increase in lipolysis or fat oxidation, effects that were described with chronic caffeine intake (45), but not to thermogenesis, since UCP1 expression in the adipose tissue was unaltered in these animals (Figure 7G). In male animals, blockade of A_2A_ and A_2B_ receptors increased total fat, which is associated with an increased in perinephric fat mass. These different effects of the adenosine receptor on weight gain and fat mass in female and male animals may be associated with hormonal and sex differences in body fat distribution and to a female higher lipolysis capacity (48, 49). Therefore, we can conclude that A_2B_ receptors are involved in the control of weight, since the blockade of these receptors increases weigh gain, especially in males, an effect that is not correlated with an increase in fat mass. We can also conclude that A_1_ receptors are involved in fat metabolism in females and A_2_ receptors in males, as blockade of A_1_ receptors decreases fat mass deposition in females and A_2A_ and A_2B_ blockade increase fat deposition in males.

### Role of Adenosine Receptors in the Control of Sympathetic Activity in Metabolic Diseases

One of the pathophysiological mechanisms described to be involved in the development of insulin resistance is the overactivation of the sympathetic nervous system (31). Herein, no sex differences were observed in the sympatho-adrenal activity in controls and HSu animals as well as in the effect of chronic administration of the different adenosine receptor antagonists in these parameters. We did not observe any significant change in circulating NE and Epi after the chronic administration of the different adenosine receptor antagonists, but the, chronic blockade of adenosine receptors increased adrenal medulla NE and Epi content in control and HSu animals (Figure 3). Under basal conditions, adenosine inhibited adrenal medulla catecholamine secretion, an effect partially achieved by the inhibitory effect of adenosine on the renin-angiotensin system and which is increased when sympathetic system is stimulated (50). Therefore, our results are in agreement with the data that shows that adenosine inhibits catecholamine secretion from adrenal medulla contrasting however with the findings that show that these effects are blunted with chronic caffeine consumption (50, 51), suggesting that the mechanisms of adaptation to caffeine are different or develop more faster than for A_1_ and A_2B_ adenosine receptor blockers. Additionally, we can suggest that the increased content of catecholamines do not mean increased adrenal medulla secretion, as we did not observe any differences in catecholamines plasma levels (Figures 3A,B) and that is not key to the maintenance of insulin resistance, as HSu animals with adenosine receptors blocked exhibit high Epi/NE adrenal medulla content but normalized levels of insulin sensitivity.

### Effect of Hypercaloric Hsu Diet on Insulin Sensitivity and Adenosine Receptor Expression

Herein we described for the first time that insulin resistance induced by HSu diet is associated with an increased expression of A_1_, A_2A_, and A_2B_ receptors in the skeletal muscle, increased expression of A_1_ in the liver and in the adipose tissue and decreased expression of A_2B_ adenosine receptors in the liver. The increase in A_1_ expression in the adipose tissue of HSu rats found in the present manuscript contrasts with the findings of Dhalla et al. (52) showing unaltered adenosine A_1_ receptor mRNA expression in Zucker diabetic fatty rat, but is consistent with the described role of A_1_ receptors in adipose tissue dysfunction (53). Also, in an obese mice model with insulin resistance induced by 16 weeks of high-fat diet, the expression of A_2B_ adenosine receptor is increased in the liver, visceral fat, and gastrocnemius muscle (54). Based on this, we can postulate that the differences in the expression of adenosine receptors might be related with differences in the animal models studied, the HSu model studied in the present work is a lean model of insulin resistance (8, 47), or with the degree of insulin resistance and disease progression, as herein we used a 4 weeks model of diet and Johnston-Cox et al. (54) submitted the animals to 16 weeks of high-fat diet. Therefore, different metabolic disturbances and different stages of disease progression might contribute differently to the expression of adenosine receptor in the insulin sensitive tissues. As expected, and consistent with the application of chronic antagonists to a system, the chronic administration of DPCPX and SCH58261 increased A_1_ and A_2A_ adenosine receptors in skeletal muscle and adipose tissue. Surprisingly, no alterations were found for the effect of adenosine antagonists in the liver and also for MRS1754, which decrease adenosine A_2B_ receptor expression in adipose tissue, without any change in skeletal muscle and liver. In HSu animals, chronic administration of SCH58261 normalized the A_2A_ adenosine receptor expression in skeletal muscle and in liver and the chronic MRS1754 administration normalized A_2B_ adenosine receptors in skeletal muscle. Interestingly, in the liver, A_2B_ adenosine receptors decreased with the HSu diet consumption, an effect that was normalized by the chronic MRS1754 administration.

We also found that the different insulin-sensitive tissues do not contribute equally or exhibit the same degree of impairment in insulin resistant states. In fact, it seems to be a notion that sometimes, and probably at the early stages of the disease, insulin resistance can be present in the absence of decreased insulin signaling [for a review see Fazakerley et al. (55)] with some authors showing evidences that the impairment in insulin action is independent of proximal elements of the insulin signaling pathway, but rather likely specific to the glucoregulatory branch of insulin signaling (55). In here, in the HSu model, obtained with 4 weeks of high-sucrose diet we found alterations in insulin signaling pathways only in the skeletal muscle, except for GLUT2 expression in the liver. One could expect also alterations in insulin signaling in the adipose tissue, but we can postulate that the mechanisms behind insulin resistance development might be different depending on the type of diet consumed and the time of exposure to the diets. In fact, it is consensual that the time-line for progression of insulin resistance in mice fed a high-fat diet starts with the development of insulin resistance in adipose tissue before the muscle (56, 57). However, the same might not be true for high sucrose diets at least when administrated during short periods of time. The liver is also another main insulin-sensitive tissue that is also involved in the development of insulin resistance (1). As previously described by our group, HSu diet did not modify extensively the expression of insulin signaling in the liver (39), since only decreased GLUT2. However, we cannot exclude possible major alterations in this tissue, as we know that insulin control of glycolysis and gluconeogenesis (1). Chronic administration of A_2_ adenosine antagonists produced the same effects in insulin signaling pathways, measured as insulin receptor expression and Glut4 transporters, in skeletal muscle, than in whole-body insulin sensitivity. Contrary results were found in the adipose tissue of control and HSu animals for chronic adenosine receptor antagonist, where the blockade of A_1_ receptors improved insulin signaling in control animals, but worsen insulin signaling in HSu animals. In contrast, no changes were found for the effect of adenosine antagonists in the liver in controls and HSu animals, suggesting that the role of adenosine in insulin action in the liver do not involve the pathways herein tested. Therefore, we can conclude that in HSu animals the effect of adenosine on insulin sensitivity is mainly mediated by A_2_ adenosine receptors in the skeletal muscle with a small contribution of A_1_ receptors in the adipose tissue.

## Conclusion

In conclusion, all together, the results herein described suggest that A_2_ adenosine receptors in the skeletal muscle are the main responsibles for the whole-body insulin sensitivity, being therefore more relevant the effect of adenosine on skeletal muscle on insulin action than the effect of adenosine on adipose tissue in a context of lean insulin resistance. We can suggest that the targeting of A_2_ adenosine receptors might be useful to rescue insulin signaling pathways in insulin-resistant conditions.

## Data Availability Statement

The datasets generated for this study are available on request to the corresponding author.

## Ethics Statement

The animal study was reviewed and approved by NOVA Medical School Ethics Committee.

## Author Contributions

JS and SC participated in research design and wrote or contributed to the writing of the manuscript. JS, FM, MR, and TR conducted experiments. JS, FM, PM, EO, and SC performed collection and data analysis. All the authors have approved the final version of the manuscript, and all persons designated as authors qualify for authorship, and all those who qualify for authorship are listed.

## Conflict of Interest

The authors declare that the research was conducted in the absence of any commercial or financial relationships that could be construed as a potential conflict of interest.
